# Self-Other Distinction Enhanced Empathic Responses in Individuals with Alexithymia

**DOI:** 10.1038/srep35059

**Published:** 2016-10-14

**Authors:** Natsuki Saito, Takemasa Yokoyama, Hideki Ohira

**Affiliations:** 1 Graduate School of Environmental Studies, Nagoya University, Nagoya, 464-8601, Japan; 2 Japan Society for the Promotion of Science, Tokyo, 102-0083, Japan

## Abstract

Although empathy is important for social interactions, individuals with alexithymia have low empathic ability, particularly where advanced empathy is concerned (empathic concern, perspective taking). It has been argued that awareness of the self-other distinction enhances advanced empathy, and alexithymics are thought to inadequately distinguish the self from others. We therefore tested whether the self-other distinction increases advanced empathy in alexithymics. To this end, we presented painful hand images over participants’ own hands, and required participants to estimate felt pain intensity and their affective states. Half of the participants got specific instructions to distinct themselves from the other in the images. Felt pain intensity (perspective taking) and other-oriented affective responses (empathic concern) were increased by the instructions only when participants had high alexithymia scores as measured by questionnaire, although self-oriented affective responses (personal distress) were not affected by the instructions. These findings indicate that enhancing the self-other distinction enhances alexithymics’ ability to use advanced empathy, but not the primitive empathy.

People often face situations that require harmonious interactions with others over the course of daily life. They often try to understand other’s mental lives, goals, and intentions in order to successfully adapt to various social conditions. Empathy is one of the essential social abilities that support smooth interactions. Empathy is applied to understand other’s emotions and to experience internal states similar to those currently being experienced by another individual^1^,^2^.

Empathy is a complex multidimensional concept, and is categorized into two factors: Affective empathy (AE) and cognitive empathy (CE)^3^,^4^,^5^. AE is defined as sharing another’s emotion during observation of the other’s emotional situation^3^. It is argued that AE occurs in parallel with the distress of the other individual and is likely to be relatively automatic^6^. In addition, it has been inferred that AE has a hierarchical structure^7^. The lower level of AE (e.g., emotional contagion) is likely developed to propel individuals frightened by the alarm of others, in order to prevent crisis due to negative arousal induced by the other’s distress calls^8^. This type of empathy is a relatively primitive process to understand others because it has been found in rats and other nonhuman social mammals^9^. The higher level of AE (e.g. empathic concern and compassion) involves a motivation toward others in need due to worry induced by the empathy, and this is associated with other-oriented and altruistic intentions and actions^10^,^11^,^12^,^13^.

On the other hand, the cognitive route to understanding others has been investigated under the guise of the Theory of Mind (ToM e.g. refs 14 and 15), which is closely related to CE. CE is a cognitive ability used to describe another’s subjective states without influence of one’s own states or biases^6^,^16^,^17^, and is associated with representation of the internal mental states of another individual^18^. CE is considered to be a more advanced process than AE, and only a few species, such as human beings, are thought to have this social ability. Such advanced empathic abilities (i.e. CE and the higher levels of AE) play key roles in fluent interpersonal interaction.

Although empathy is an essential social ability to understand others, there are large individual differences in degree of empathic ability, such that some have less empathic ability than others. Alexithymia appears to be one trait associated with such a difference^19^,^20^,^21^. People with alexithymia show deficits in describing and recognizing emotions within themselves, in distinguishing feelings from the bodily sensations of emotional arousal, and are preoccupied by internally oriented thinking^22^. Previous studies of dispositional empathy, as measured by the Interpersonal Reactivity Index (IRI^3^,^23^), have shown that individuals with alexithymia do not differ from others in low level AE, whereas they do evidence lower scores on “perspective taking” and “empathic concern”, which are related to CE and high level AE respectively^20^,^21^,^24^. In addition, a previous study has indicated that alexithymic individuals show fewer altruistic behaviors^25^ and more utilitarian tendency in judgement about moral dilemma^26^,^27^,^28^, compared with non-alexithymic persons. Despite intact primitive AE abilities, individuals with alexithymia appear to struggle with more sophisticated applications of empathy for others.

Even though previous studies have indicated that people with alexithymia have less advanced empathic abilities than people without alexithymia^20^,^21^,^24^,^25^, it is conceivable that such individuals could display relatively advanced empathy. A key possible factor concerns the self-other distinction. A previous evolutionary psychology review has suggested that achievement of empathy depends on increasing the self-other distinction, because understanding individual’s emotional states would of course require one to attribute such a state to another individual, at least initially, a feat that requires a shift to an other-oriented perspective^7^,^29^,^30^. Moreover, previous empirical studies have found that when participants (non-alexithymic) are more conscious of the self-other distinction (e.g., via training), their advanced empathic responses are improved^31^,^32^. This enhancement of empathy has found in both explicit and implicit indices, and lasts for at least 24 hours after training^33^. Importantly, alexithymic individuals have deficits in distinguishing between self and other^34^. Given these findings, we hypothesized that people with alexithymia might display advanced empathy when they are more conscious of the self-other distinction.

The aim of this study was to investigate whether the advanced empathic responses of alexithymic people can be raised by enhanced awareness of the self-other distinction. To this end, we attempted to manipulate the self-other distinction using simple verbal instructions. Color images depicting the painful right hand of another individual were presented superimposed over participants’ right hands, in the form of the first-person image (Fig. 1). Half the participants were assigned to a self-other distinction group, such that they were explicitly instructed to view the picture as the hand of another individual. The other half were assigned to the passive observing group, and were instructed to observe the image passively without any instructions regarding perspective. We measured the empathic response to another’s pain via ratings of felt pain when participants saw the painful pictures, and participants also rated their own affective responses to the stimuli. This paradigm permitted us to test the novel suggestion that individuals with alexithymia are in fact able to cognitively empathize with others. Such a finding may indicate that empathy deficits in alexithymic people are related to deficits in spontaneous adoption of the self-other distinction.

## Result

### Alexithymia scores’ moderation effects for the association between empathy and self-other distinction

We investigated whether consciousness of the self-other distinction increased advanced empathic responses in individuals with alexithymia. We employed stepwise multiple regression analyses in order to determine the moderation effects of the Gotow Alexithymia Questionnaire (Galex^35^) for the relationship between empathy and specific instructions regarding the self-other distinction. We entered instruction (observing the other’s pain according to a clear self-other distinction =1, passive observing = 0) and alexithymia tendency assessed via Galex scores in Step 1, and their interaction (instruction × Galex) in Step 2. Before creating the interaction terms, all variables were centered^36^. We investigated relationships between alexithymia tendency and empathy scores in terms of felt pain intensity, other-oriented affective responses, and self-oriented affective responses. Simple slope analysis was adopted as the sub effect test to examine the effect of instructions, with mean Galex score ± 1*SD*^37^. The regression analysis results are summarized in Table 1.

### Pain intensity

In Step 1, the main effects of instruction (*β* = 0.06, *p* = 0.61) and Galex (*β* = 0.21, *p* = 0.07) were not significant. In Step 2, there were no main effects of instruction (*β* = 0.05, *p* = 0.66) or Galex score (*β* = −0.61, *p* = 0.11), but there was a significant instruction × Galex interaction, (*β* = 0.86, *p* = 0.03; Interaction effect *R*^*2*^ change = 0.11, *p* = 0.03). Simple slopes analysis denoted that the effect of instruction was significant for high alexithymia individuals (*β* = 0.30, *p* = 0.03) but not for those low in this trait (*β* = −0.20, *p* = 0.21). Thus, whereas low alexithymia individuals were unaffected by the instruction—implying that they were already taking the other’s perspective— high alexithymia individuals estimated significantly higher pain intensity when they had been instructed to distinguish others from themselves, as compared with no such instructions (Fig. 2A).

### Affective response

In Step 1, we found no significant main effects of instruction (*β* = 0.12, *p* = 0.30) or Galex (*β* = −0.09, *p* = 0.45) on other-oriented affective responses. In Step 2, there was no main effect of instruction (*β* = 0.11, *p* = 0.33), but there was a significant main effect of Galex score (*β* = −0.91, *p* = 0.02) and an instruction × Galex interaction, (*β* = 0.86, *p* = 0.03, Interaction effect *R*^*2*^ change = 0.06, *p* = 0.03). Simple slopes analysis indicated that the instruction effect was significant for high alexithymia individuals (*β* = 0.36, *p* = 0.02) but not for individuals with low alexithymia (*β* = −0.14, *p* = 0.39). High alexithymia individuals provided significantly higher other-oriented affective responses when they had (vs. had not) been instructed to distinguish others from themselves (Fig. 2A).

Regarding self-oriented affective responses, in Step 1 there were no significant main effects of instruction (*β* = 0.18, *p* = 0.11) or Galex (*β* = 0.01, *p* = 0.95). In Step 2, there was no main effects of instruction (*β* = 0.18, *p* = 0.11) or Galex score (*β* = 0.02, *p* = 0.95), and a non-significant instruction × Galex interaction, (*β* = −0.02, *p* = 0.97, Interaction effect *R*^*2*^ change = 0.00, *p* = 0.97). These results suggest that the instruction to distinguish others from themselves did not influence primitive AE, which seems to have been generated immediately (Fig. 2A).

## Discussion

We investigated whether highlighting the self-other distinction works to enhance the advanced empathic responses of individuals with alexithymia. To address this question, we provided instructions that required participants to distinguish themselves from another’s hand in the presented pictures. Participants estimated felt pain intensity of other’s painful hand images presented over participant’s hand and answered their own affective states after observation. Estimated pain intensity (i.e., perspective taking) and other-oriented affective response (i.e., empathic concern) were enhanced by the instructions only in individuals with alexithymia, although self-oriented affective responses (i.e., personal distress) was not affected by the instruction regardless of alexithymia score. Emphasizing the self-other distinction enhanced relatively advanced empathic responses in alexithymics, but not more primitive ones.

The present study provides the first evidence that conscious awareness of the self-other distinction improves empathy in alexithymia. Although a previous study indicated that people with alexithymia display deficits in advanced empathy^20^,^21^,^24^,^25^, our data suggest that such individuals can in fact display advanced empathy when faced with another’s pain, assuming that they are conscious of the self-other distinction. In terms of an evolutionary theoretical perspective, de Wall (2008) has suggested that perspective taking and empathic concern accompany increased salience of the self-other distinction, such that when typical individuals observe another’s emotional situation, they must attribute their own emotional state to the experience of the other, if empathic responding is to occur. Awareness of the self-other distinction does appear to enhance advanced empathy^31^,^32^,^33^. Although a previous study indicated that individuals with alexithymia show deficits in making the self-other distinction^34^, they might be just not good at distinguishing self from others. If they are provided some cues regarding self-other distinction, their self-other distinction functions well.

It is conceivable that alexithymic individuals might fail to acquire the ability to automatically apply the self-other distinction during their development. This might provoke impaired emotional self-consciousness and consequently poor advanced empathy abilities. Although self-other distinction is usually acquired thought development^38^, alexithymic individuals tend to have deficits in self-consciousness of emotion during their development^39^,^40^. For instance, childhood abuse disturbs development of recognizing and labeling emotional states, and such childhood experiences are more likely to produce alexithymic characteristics^39^. Because the self-other distinction and self-consciousness of emotion are closely associated^41^, developmental disturbance of the latter should lead to similar disturbance of the former. In fact, alexithymic individuals show deficits in both making the self-other distinction and self-consciousness of emotion^22^,^34^,^42^. However, in our study, participants whose alexithymic scores were high showed advanced empathy when they were instructed to have a strong sense of the self-other distinction. Thus, while self-other distinction is not completely impaired in people with alexithymia, they probably cannot use this ability automatically or unconsciously. Future work is required to test this possibility.

We found that evoking use of the self-other distinction did not enhance primitive empathy (i.e., personal distress) in alexithymia. This is also consistent with previous studies. Several previous studies have indicated that although individuals with alexithymia show no deficits in primitive empathy, advanced empathy is impaired^20^,^21^,^24^,^25^, but see Bernhardt *et al*.^43^. Given this pattern, we did not find any instruction-related differences in self-oriented affective scores between the low and high alexithymics.

Our finding of no effect of instructions on low alexithymia individuals is not consistent with previous studies that examined healthy samples^32^,^33^. In the previous studies, empathy training was implemented. Perhaps an effect was observed in this study because training induces strong learning effects regarding the particular training. We focused on participants’ awareness of the self-other distinction exactly when they observe and estimate another’s pain, essentially asking participants to use what they already know on some level, whereas the previous studies examined unconscious improvement self-other awareness as a function of training. We focused on participants’ awareness of self-other distinction at their observation and estimation time, whereas the previous studies focused on unconscious improvement of ability about self-other distinction via training. We needed to avoid training that induces training-specific effects and to use the instruction as easy and instantaneous ways. Furthermore, Lamm *et al*.^31^ compared participants who attempted to the other’s painful facial expressions as the other (taking other-perspective) with who attempted to the images as self (taking self-perspective). We avoided use of self-perspective instructions in our experiment. Lamm *et al*.^31^ indicated that taking the self-perspective decreased empathic concern in non-alexithymics, compared to an other-oriented perspective. Because individuals with alexithymia have low self-conscious awareness of emotion^22^,^42^,^44^, their self-consciousness of emotion should be fragile and relatively susceptible to disturbance via various factors. Taking the self-perspective might alter awareness of emotional self-states in alexithymics due to an increased self-focus of attention, because taking self-perspective might induce self-attention. When people are required to focus on their own representation, this induces their self-attention^45^,^46^,^47^, so it can alter awareness of emotional self-consciousness in alexithymics. The aim of our study was to observe relatively pure effects of the self-other distinction, such that we did not use self-perspective as a control condition. It is conceivable that passive viewing (our study) and adopting a self-perspective via instructions (the study of Lamm and colleagues) elicit large differences about empathic scores in non-alexithymics, and the differences between comparisons regarding perspectives might induce the inconsistency.

In conclusion, we investigated whether using instructions to highlight the distinction between self and other might enhance high alexithymic individuals’ ability to use advanced empathy. We found that this is indeed the case, suggesting that the self-other distinction plays an important role in inducing more advanced cognitive empathy in alexithymia, although more primitive empathic processes seem to be intact in such individuals. Future studies should investigate whether effects of highlighting the self-other distinction persist for longer durations post-experiment. Persistence of such effects would be quite relevant to clinical intervention.

## Method

### Participants

Seventy-eight undergraduates participated in this study (mean age = 20.50 ± 1.36 years, 76 right-handed, 21 men). All participants gave their informed written consent. The procedure was conducted in accord with the Declaration of Helsinki.

### Gotow Alexithymia Questionnaire

We used the Gotow Alexithymia Questionnaire (Galex^35^) to measure alexithymia in this study although Toronto Alexithymia Scale (TAS-20) has been more commonly used to assess alexithymia^48^,^49^. Both Galex and TAS-20 have structural factors of “difficulty identifying feelings”, “deficits in describing feelings”, and “externally oriented thinking”, but only Galex has “lack of imagination” as a structural factor. In the current study, participants saw a painful picture and needed to “imagine” how painful it was, so Galex that could measure lack of imagination was more appropriate in our study. Also, Galex has good test-retest reliability and internal consistency^35^. Therefore, we selected to use Galex, instead of TAS-20, in our study.

Galex has a four-factor structure. The first factor of Galex concerns difficulty identifying feelings and bodily sensations (e.g. “I am often confused about what emotion I am feeling”). The second is deficits in describing feelings and bodily sensations (e.g. “I find it hard to describe how I feel about people”). The third is a specific tendency to deal with superficial themes but not more deep affective thinking (e.g. “I am not willing to know why something happened of my own accord”). The fourth is lack of imagination (e.g. “I daydream rarely”). Responses were rated on a seven-point Likert scale (1 = certainly does not apply to me, 7 = certainly does apply to me), and higher scores indicated more pronounced alexithymic characteristics. To confirm its reliability and factor structure, we administered the Galex to another group of participants (N = 256), and conducted factor analysis via the maximum-likelihood method using Promax rotation. We confirmed a four-factor structure. These factors were largely congruent with those obtained by Gotow *et al*.^35^. The first factor is “deficits in identifying and expressing one’s own affect and physical sensation (α = 0.80)”, the second factor is “deficits in fantasy and introspection (α = 0.79)”, the third factor is “Externally oriented thinking (α = 0.77)” and the fourth factor is “Lack of fantasy (α = 0.64)”. Mean total Galex score was 58.4, and the standard deviation was 9.17. We used total Galex score in our analysis.

### Picture stimuli

We prepared a series of 26 digital color pictures that depicted human right hands under painful situations. Those pictures were taken from the first-person angle (no mental rotations were required for the observer), as referenced in a previous study^50^. Various types of pain (injection, cutting, burning) were represented in commonly occurring situations. The painful pictures used in this study were selected based on our preliminary investigation (N = 10; mean age = 21.3 ± 0.64 years, 5 men). Participants in the preliminary investigation estimated how painful the depicted situations would be. We used pictures that averaged an intensity of felt pain of greater than 1.00 (0: No Pain to 3: Worst unbearable pain).

### Affective response questionnaire

We used the affective response questionnaire referring to in previous research^51^. We adopted 19 items, omitting one unsuitable item that does not bear upon painful situations. There were three subscales: Parallel affective response (7 items), unpleasant response (3 items), and other-oriented response (9 items). The first two subscales assessed self-oriented affects. Examples of questions in those two scales were “I feel pain looking at that” and “I’m shaking” The other-oriented response scale evaluated other-oriented affects and related to altruism, including items such as “I’d like to help them” and “I feel sorry for them” We used self-oriented response and other-oriented response scores as measures of primitive AE and advanced AE, respectively. Other-oriented responses provoke an altruistic motivation to help the other, whereas self-oriented responses are related to egoistic behavior to reduce shared distress^1^,^2^. A previous review mentioned that these two types of affective responses may often occur at the same time, but are nevertheless distinct^52^. Responses were rated on a seven-point Likert scale (1 = certainly does not apply to me, 5 = certainly does apply to me).

### Procedure

**Figure 1:**
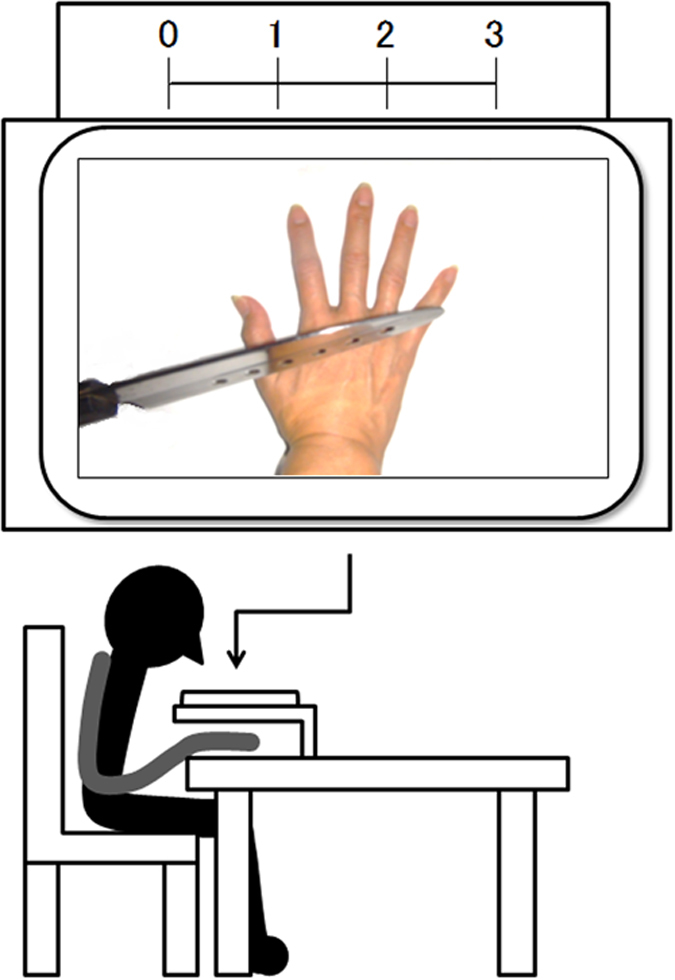
Sample pictures of painful hands and presentation method.

**Figure 2:**
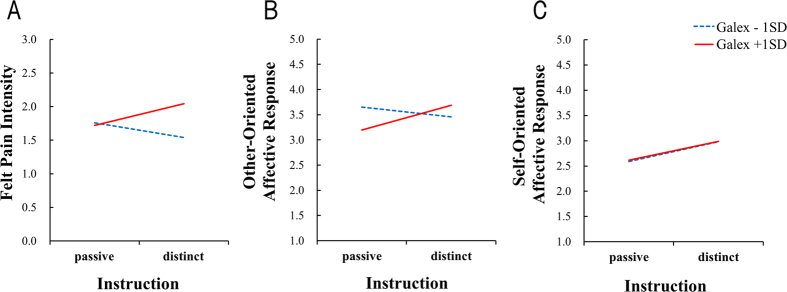
When alexithymia score was high, instructions affected (**A**) estimation of other’s pain intensity and (**B**) self-reported other-oriented affective response, but not (**C**) self-reported self-oriented affective response.

Participants were presented color photographs depicting human right hands were injured by various tools (e.g. a knife, cutter, needle, cigarette lighter). These stimuli were superposed over participants’ right hands using a 4th generation iPad, in a first-person orientation (Fig. 1). Participants were divided into self-other distinction and passive observer groups. Participants in the self-other distinction group attempted to see the pictures of the right hand as another’s with the specific instruction that is “Please imagine that the pain is not yours, but rather that of another when you assess the pain intensity of the pictures”, whereas participants in the passive observer group were instructed to observe the images passively and assess the felt pain intensity. All participants were asked to evaluate pain intensity from 0 (no pain) to 3 (worst unbearable pain) for each image. Each painful image was presented for 4 s after a 1 s fixation presentation. Ratings occurred after presentation of a fixation point. After the pain-assessing task, affective responses were measured via the affective response questionnaire. After the experiments, participants in the self-other distinction group were asked if they were able to look at the picture stimuli as the hand of another. Conversely, participants in the passive observer group were asked if they thought of the pain as that of another or themselves. We rejected the data of participants in the passive observer group who provided pain ratings based on those of another.

## Additional Information

**How to cite this article**: Saito, N. *et al*. Self-Other Distinction Enhanced Empathic Responses in Individuals with Alexithymia. *Sci. Rep*. **6**, 35059; doi: 10.1038/srep35059 (2016).

## Figures and Tables

**Table 1:** Regression analysis results.

Variable	Felt Pain Intensity				Other-Oriented Affective Response				Self-Oriented Affective Response			
	Step 1		Step 2		Step 1		Step 2		Step 1		Step 2	
	*B*	*SE*	*B*	*SE*	*B*	*SE*	*B*	*SE*	*B*	*SE*	*B*	*SE*
Step 1												
Instruction	0.06	0.12	0.05	0.12	0.17	0.16	0.15	0.16	0.39	0.24	0.39	0.25
Galex	0.19	0.10	−0.53	0.33	−0.10	0.13	−1.02*	0.43	0.01	0.19	0.04	0.67
Step 2												
Instruction × Galex			0.57*	0.20			0.57*	0.25			−0.02	0.40
Δ*R*^*2*^	0.05		0.06*		0.02		0.06*		0.03		0.03	
* Adj R*^*2*^	0.02		0.07		0.00		0.05		0.01		0.00	

*p < 0.05.
